# A *dystroglycan–laminin–integrin* axis coordinates cell shape remodeling in the developing *Drosophila* retina

**DOI:** 10.1371/journal.pbio.3002783

**Published:** 2024-09-03

**Authors:** Rhian F. Walther, Courtney Lancaster, Jemima J. Burden, Franck Pichaud

**Affiliations:** Cell Biology of Tissue Architecture and Physiology. Laboratory for Molecular Cell Biology (LMCB), University College London, London, United Kingdom; King’s College London, UNITED KINGDOM OF GREAT BRITAIN AND NORTHERN IRELAND

## Abstract

Cell shape remodeling is a principal driver of epithelial tissue morphogenesis. While progress continues to be made in our understanding of the pathways that control the apical (top) geometry of epithelial cells, we know comparatively little about those that control cell basal (bottom) geometry. To examine this, we used the *Drosophila* ommatidium, which is the basic visual unit of the compound eye. The ommatidium is shaped as a hexagonal prism, and generating this 3D structure requires ommatidial cells to adopt specific apical and basal polygonal geometries. Using this model system, we find that generating cell type–specific basal geometries starts with patterning of the basal extracellular matrix, whereby Laminin accumulates at discrete locations across the basal surface of the retina. We find the Dystroglycan receptor complex (DGC) is required for this patterning by promoting localized Laminin accumulation at the basal surface of cells. Moreover, our results reveal that localized accumulation of Laminin and the DGC are required for directing Integrin adhesion. This induces cell basal geometry remodeling by anchoring the basal surface of cells to the extracellular matrix at specific, Laminin-rich locations. We propose that patterning of a basal extracellular matrix by generating discrete Laminin domains can direct Integrin adhesion to induce cell shape remodeling in epithelial morphogenesis.

## Abstract 2

Cell shape remodeling is a main driver of epithelial tissue morphogenesis, but how cell basal geometry is regulated remains unclear. This work identifies a novel morphogenetic pathway involving laminin and integrin adhesion that coordinates the remodelling of epithelial cell shape by patterning their basement membrane.

## Introduction

The function of most organs depends on epithelial tissues. In these tissues, cells coordinate their polarity to generate distinct apical and basal surfaces. This organization underpins epithelial tissue function in polarized transport of macromolecules or gas across compartments. At the apical surface, cells adhere to one another through apical-lateral junctions, which seal the epithelial barrier [1]. Basally, cells are attached to the basement membrane, which, among other functions, provides biomechanical support to tissues [2]. In addition to this apical-basal organization, epithelia are often organized in 3 dimensions, including folds and curved regions, which are essential for generating physiological compartments. Generating 3D tissue organization requires groups of cells to coordinate changes in their position and shape as tissues develop. These changes have been mostly studied in 2 dimensions, focusing on the apical surface of tissues. We know comparatively little about how the basal surface of a tissue is remodeled through development.

Most studies examining cell movement and shape remodeling during epithelial tissue development tend to make use of embryonic tissues, which consist of a homogenous cell population. This approach has proven extremely valuable to reveal fundamental processes in epithelial patterning and morphogenesis. For example, the relative movement of cells in the plane of a tissue can promote tissue elongation [3–7]. Moreover, changes in cell apical or basal surface area can contribute to inducing tissue curvature, ranging from fold and tube formation [8–15] to tissue invagination [16–19]. In principle, any polygonal geometry can be generated at the apical surface of epithelial cells, depending on the number of adherens junctions (i.e., number of neighbors) and junction length. As these homogenous tissues mature, cells often adopt a hexagonal geometry at their apical surface to minimize surface energy [20]. The situation is likely to be more complex in tissues where cells acquire specific shapes as they undergo differentiation. A good example of this is found in the *Drosophila* eye. In this sensory epithelium, each of the 3 epithelial cell types that make up the apical surface (lens) remodel their geometry to adopt stereotypical shapes. The combination of programed cell death, preferential adhesion between different cell types, and actomyosin regulation contributes to determining these different apical geometries [21–30].

Alongside these apical regulations, cells can remodel their basal surface [7,31,32]. Basally, cells are attached to a basement membrane via cell–ECM adhesion molecules such as Integrins. Integrins are heterodimers consisting of an α and β chain, which can bind to extracellular matrix (ECM) components of the basement membrane. Integrin ligands include Laminins [33,34] and Collagen IV [35,36], which are both large trimeric proteins. Integrin binding to these ECM components connects the basement membrane to the cell’s cytoskeleton through multiple adaptor proteins such as Talin and ILK [37–39]. In *Drosophila*, Integrin receptors include 5 possible alpha chains (αPS1/Mew, αPS2/if, αPS3/Scb, ItgaPS4, and ItgaPS5) and 2 beta chains, βPS1/Mys and Itgbn/βv [40]. In developing epithelia, Integrins have been shown to regulate the basal area of cells. This is the case in the follicular epithelium of the *Drosophila* ovary, where Integrins organize the basal F-actin cytoskeleton [41,42]. In this epithelium, another basal surface receptor, the Dystroglycan receptor complex (DGC), which consists of Dystroglycan (Dg), Sarcoglycan subunits α, β, and δ, and the adapter protein Dystrophin, also contributes to organizing the basal F-actin cytoskeleton [43,44]. The DGC can bind to Laminins [45], and its function in organizing basal F-actin in follicular cells depends upon the formation of Laminin fibrils in the underlying basement membrane [43]. Interestingly, work using cultured mouse embryonic stem cells has also revealed a requirement for DG in organizing Laminin-1 within the extracellular matrix during embryoid body formation [46]. Moreover, Dg overexpression in the developing *Drosophila* trachea leads to a precocious accumulation of Laminin at the basal surface of the tracheal cells [47]. Thus, Laminin organization and the DGC are intimately linked in basement membrane development, and this relationship appears to be conserved through evolution. How basement membrane regulation, the DGC, and Integrins might regulate tissue basal surface remodeling in epithelial morphogenesis largely remains to be investigated.

To examine this gap in knowledge, we made use of the genetically amenable *Drosophila* retina. This sensory epithelium is made of 750 basic visual units called ommatidia. Each ommatidium is shaped as a hexagonal prism, and within this 3D structure, different cell types adopt specific apical and basal polygonal geometries [48]. Using this model system, we show that coordinating changes in cell basal geometry across groups of cells involves a switch in Integrin localization, from a basal cluster to polarized localization. We find that this polarized Integrin accumulation at the basal surface of cells is directed by Laminin, which accumulates at discrete locations across the ECM that lines the basal surface of the epithelium. Furthermore, we present evidence that the DGC is required to promote this localized Laminin accumulation. We conclude that during epithelial tissue development, patterning of the ECM through the DGC and Laminins can coordinate cell shape remodeling by directing the location of Integrin at the cells’ basal surface.

## Results

### Retinal cells remodel their basal geometry to shape the ommatidium as a hexagonal prism

**Fig 1:**
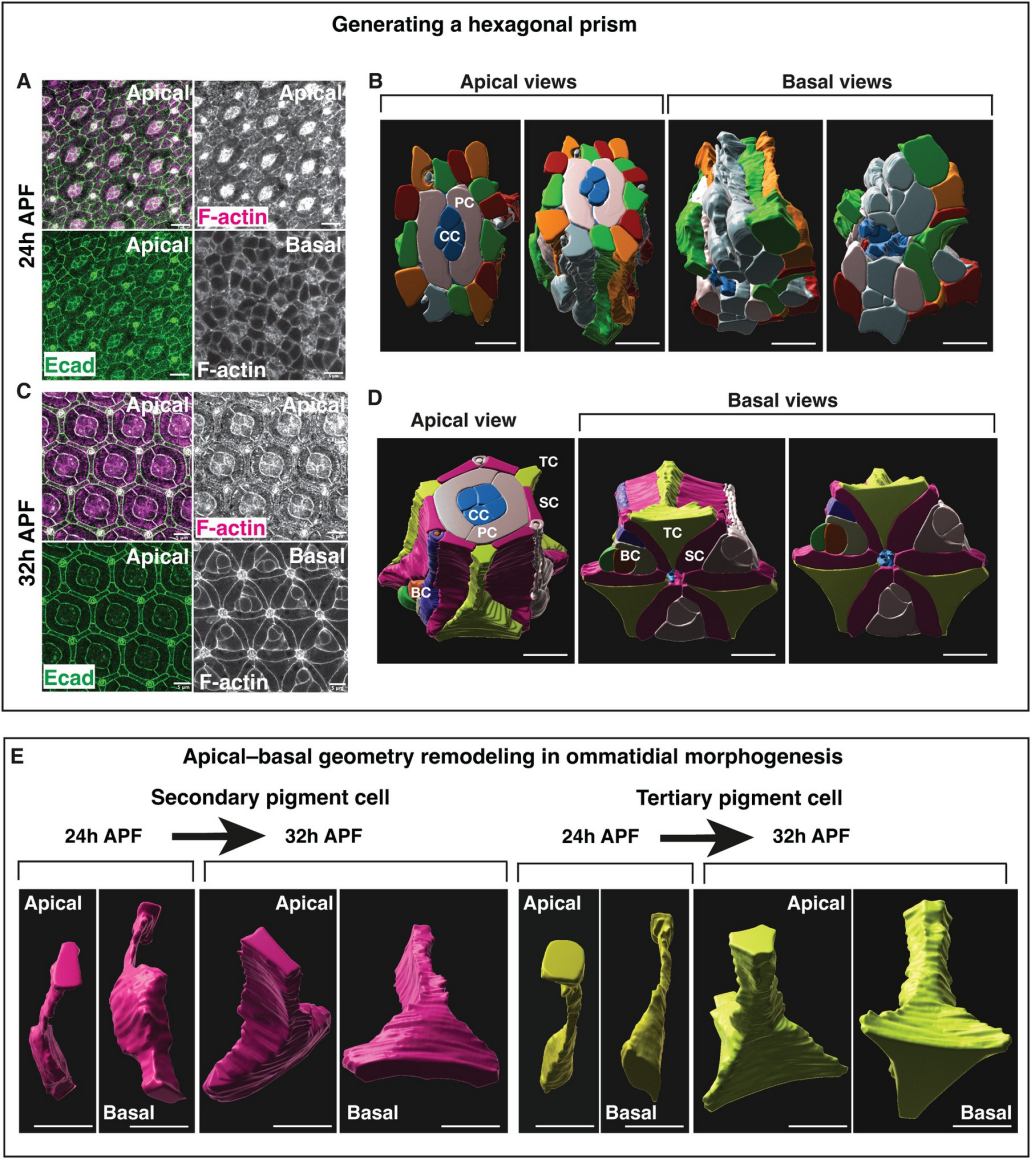
Retinal cells acquire specific basal geometries in morphogenesis. **(A)** Confocal images of the apical and basal surfaces of an early pupal retina (24h APF). F-actin (magenta) and Ecadherin::GFP labels the apical Adherens Junction (AJ) (green). **(B)** 3D segmentation and rendering of an early ommatidium (24h APF), showing apical and basal side views. The cone cells (CC) at the center of each ommatidium are labeled in blue. Primary pigment cells (PC) are labeled in light grey. The other cells are labeled with random colors to help track them along the apical-basal axis. **(C)** Confocal images of the apical and basal surface of the same patterned pupal retina (32h APF). F-actin (magenta) and Ecadherin::GFP labels the AJ (green). **(D)** 3D segmentation and rendering of a patterned ommatidium (32h APF) showing apical and basal side views. Cone cells (CC, blue), primary pigment cells (PC, grey), secondary pigment cells (SP, magenta), tertiary pigment cells (TC, yellow), and bristle cell complex, which consist of 4 cells (BC). Scale bars: 5 μm. **(E)** Individual secondary (magenta) and tertiary (yellow) before (24h APF) and after (32h APF) geometry remodeling. Scale bars: 5 μm.

To characterize cell basal geometry remodeling in the retina, we made use of confocal microscopy to generate 3D segmentations of the ommatidial cells as they remodel their shape. During early ommatidial morphogenesis, cells present poorly defined apical and basal geometries (Fig 1A and 1B and S1 and S2 Movies). As ommatidial morphogenesis proceeds, cells progressively remodel their apical and basal geometries to shape the ommatidium as a hexagonal prism (Fig 1C and 1D and S3 and S4 Movies). Basally, the secondary pigment cells adopt an oblong geometry, and the tertiary pigment cells adopt a triangular geometry [48] (Fig 1E). The bristle cell complex, which consists of 4 cell types, forms a triangular shape, which alternates with the tertiary pigment cells, in between secondary pigment cells (Fig 1C and 1D). At the tissue level, the secondary and tertiary pigment cells, which contribute to 2 and 3 neighboring ommatidia, respectively, generate a supracellular lattice that connects ommatidia across the basal surface of the epithelium (Figs 1C and S1).

### Integrins become polarized during cell basal geometry remodeling

**Fig 2:**
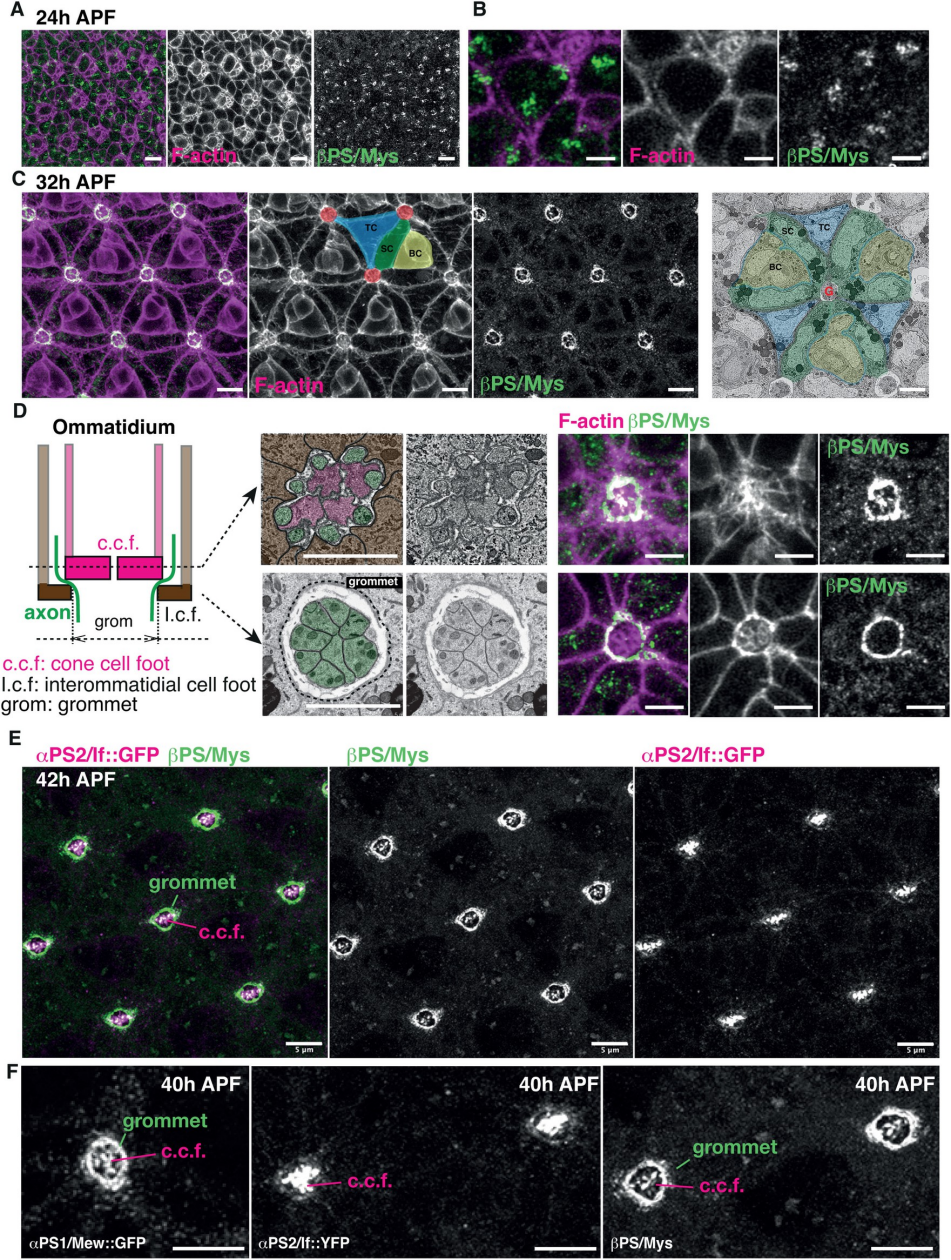
Integrins become polarized as cells remodel their basal geometry. **(A)** Basal surface of a 24h APF retina, before cell basal geometry remodeling has occurred. F-actin (magenta) and Mys/βPS (green). **(B)** Higher magnification of the basal surface of retinal cells prior to geometry remodeling. **(C)** Basal surface of a 32h APF retina, as cells have acquired their basal geometry, F-actin (magenta) and βPS/Mys (green). One secondary (SC, green) and one tertiary (TC, blue) pigment cell is highlighted to show how these cells connect multiple neighboring ommatidia. The center of the ommatidia that they connect are labeled in red. A bristle cell complex (BC) is highlighted in yellow. One electron micrograph of a basal section of the retina is shown, centered on one ommatidium. SC: secondary pigment cells (green), TC: tertiary pigment cells (blue), BC: Bristle cell complex (yellow). Grommet structure (G) around the afferent photoreceptor axons (a). **(D)** Simplified (2D) drawing of a sagittal section of the ommatidium, showing 2 of the 4 cone cell feet (c.c.f, magenta) and 2 of the 6 interommatidial cell feet (i.c.f, brown). The afferent photoreceptors axons (green) run at the periphery of the c.c.f and exit the ommatidium in between the i.c.f. The grommet structure is delineated by the plasma membrane of the pigment cells, which surround the c.c.f and axons. The electron micrographs show a distal cross sections cutting through the feet of the cone cells (magenta), the axons (green), and the interommatidial cells (brown), and a more proximal section cutting through the axons only, below the feet of the cone cells. The confocal staining shows optical cross sections at this location, showing that βPS/Mys accumulates at the feet of the cone cells and at the grommet. **(E)** Basal surface of a 42h APF retina αPS2/If::GFP (magenta) and βPS/Mys (green). The αPS2/If::GFP panel shows how this subunit is detected at the c.c.f, and not at the grommet. **(F)** Projection of confocal section spanning the c.c.f and grommet focal plans. Note how the cone cells express both αPS1/mew and αPS2/if, while the pigment cells delineating the grommet only express αPS1/mew. Scale bars **(A, C, E, F):** 5 μm and **(B, D):** 2 μm.

To understand how cell basal geometry remodeling is induced, we sought to identify gene requirements in this process. The Integrin *βPS* subunit (Myospheroid (Mys)) is required to maintain surface integrity late in retinal development, as the tissue surface undergoes basal contraction [48,49]. Here, we asked whether Integrin adhesion is also required for cell basal geometry remodeling, which precedes contraction. Monitoring the expression of the βPS/Mys subunit revealed that before cell basal geometry remodeling begins, Integrins concentrate in clusters at the basal surface of cells (Fig 2A and 2B). As interommatidial cells remodel their basal geometry at around 32h after puparium formation (APF), βPS/Mys becomes polarized at their basal surfaces. This polarization leads to the accumulation of βPS/Mys around the center of the ommatidium (Fig 2C). This generates what has been referred to before as the grommet, a supracellular structure anchoring the interommatidial cells around the basal feet of the cone cells and the afferent photoreceptor axons [48] (Fig 2C and 2D). Distally, βPS/Mys accumulates at the plasma membrane of the interommatidial cells, where they encircle the feet of the cone cells. βPS/Mys also accumulates at the basal surface of the cone cells (Fig 2D). Proximally, βPS/Mys continues to accumulate at the plasma membrane of interommatidial cells to form a supracellular ring of Integrin adhesion around the photoreceptor axons and cone cell feet (Fig 2D). To confirm that the Integrin staining at the grommet is contributed by the interommatidial cells, we generated retinas mosaic for *talin* (*rhea*) RNAi, an essential component of the Integrin adhesion complex. In secondary and tertiary pigment cells expressing *talin* RNAi, βPS/Mys localization at the grommet was lost, demonstrating that βPS/Mys accumulation at the grommets results from the polarization of Integrins in the interommatidial cells (S2A and S2B Fig). Expanding our survey of Integrin expression to the α chains, αPS1 (Multiple edematous wing (Mew)) and αPS2 (Inflated/If) revealed that the cone cell feet present both these αPS chains (Figs 2E and S3). In contrast, within the grommet, the interommatidial cells only present αPS1/Mew (Figs 2F and S3). Therefore, different retinal cell types present different combinations of α and β Integrin subunits.

### Integrins are required for cell basal geometry remodeling

**Fig 3:**
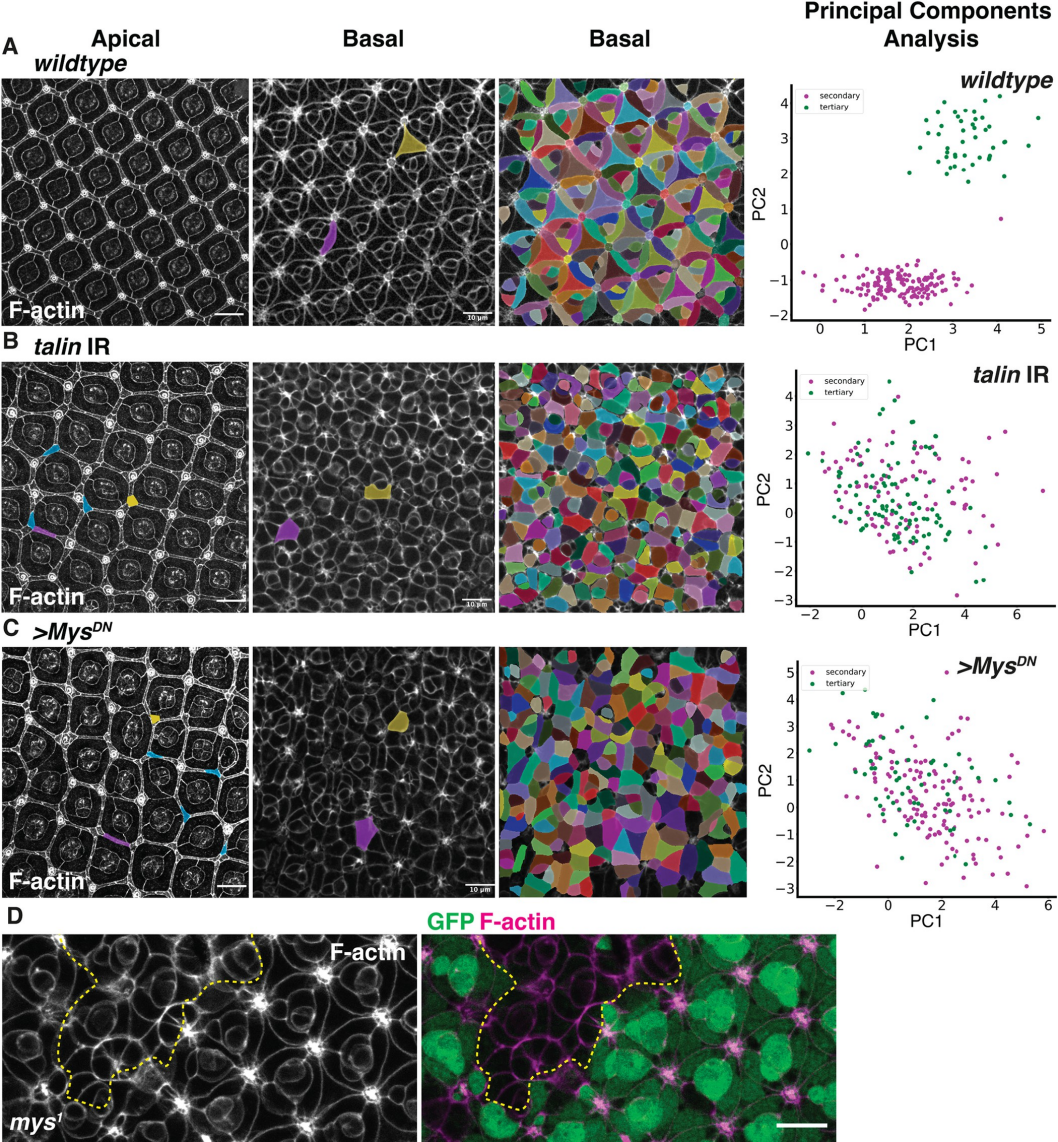
Integrin adhesion is required for cell basal geometry remodeling. **(A) From left to right:** Confocal section of a wild-type patterned retinal apical surface at 42h APF (secondary cell in magenta and tertiary cell in yellow). Confocal section of the basal surface from the same retina (secondary cell in magenta and tertiary cell in yellow). Segmentation of this basal surface using napari and processed through a PCA allowing us to distinguish the basal geometry of the secondary (magenta) and tertiary (green) pigment cells. **(B)** Apical section of a GMR-Gal4, *talin* RNAi retina at 42h APF (secondary cell in magenta and tertiary cell in yellow). Confocal section of the basal surface from the same retina (secondary cell in magenta and tertiary cell in yellow, undetermined cell in blue). Segmentation of this basal surface using napari and processed through a PCA allowing us to distinguish the basal geometry of the secondary (magenta) and tertiary (green) pigment cells. **(C)** Apical section of a GMR-Gal4, *Mys*^*DN*^ retina at 42h APF (secondary cell in magenta and tertiary cell in yellow). Confocal section of the basal surface from the same retina (secondary cell in magenta and tertiary cell in yellow, undetermined cell in blue). Segmentation of this basal surface using napari and processed through a PCA allowing us to distinguish the basal geometry of the secondary (magenta) and tertiary (green) pigment cells. **(D)** Basal optical section of a *mys*^*1*^ mutant clone. Tissue lacking GFP is circled using a yellow dashed line. F-actin (magenta) GFP (green). Scale bars: 10 μm. Data associated with figure panels **(A-C)** can be found in S1 Data.

Next, we examined the requirement of Integrins in retinal cell shape remodeling. To do this, we expressed a *talin* RNAi and a dominant negative *Mys*^*DN*^ transgene that allows for Integrin signaling but abolishes binding to ECM components [50]. We also made use of the *mys*^*1*^ loss-of-function allele. We expressed *talin RNAi* and *Mys*^*DN*^ transgenes in all retinal cells using the GMR-Gal4 driver [51]. To examine the basal geometry of the retinal cells in a quantitative manner, we used automated segmentation with manual correction [52] to quantify multiple cell shape parameters at the basal surface, including area, perimeter, and circularity. We focused our analysis on the secondary and tertiary pigment cells, and we used a principal component analysis (PCA) to understand the major axes of variation between cells. This analysis allowed us to distinguish between the secondary and tertiary pigment cells in wild-type retina (Fig 3A). Using this methodology, we found that inhibiting *talin* expression leads to a highly perturbed basal surface compared to wild type. In these retinas, the basal geometry of secondary and tertiary pigment cells was undistinguishable from each other (Fig 3B). Similar to *talin* RNAi, expressing the *Mys*^*DN*^ transgene disrupts basal surface organization, as the basal geometry of the secondary and tertiary pigment cells could no longer be distinguished (Fig 3C). Further, generating *mys*^*1*^ mutant clones leads to a cell-autonomous disruption of cell basal geometry, like that observed in *talin* RNAi and Mys^DN^ retina (Fig 3D).

To understand which parameters explained most of the variance in the PCA, we generated correlation circle plots (S4 Fig). We found that for wild-type cells, perimeter and circularity contributed most to the variance between secondary and tertiary pigment cells along the PC1 axis. Eccentricity and minor axis length contributed most to the variance along the PC2 axis (S4A Fig). For *talin* RNAi and *Mys*^*DN*^ cells, the correlation circle plots were remarkably similar (S4B and S4C Fig), indicating that these genetic perturbations had similar effects on cell basal geometry. To confirm this result, we performed PCA comparing secondary and tertiary pigment cells for these 2 genotypes. In both genotypes, cells failed to form discrete clusters (S4D and S4E Fig). For the secondary pigment cells, expressing *talin* RNAi or *Mys*^*DN*^ led to an increase in cell roundness, while for the tertiary pigment cell, these genotypes lead to an increase in circularity (S4F and S4G Fig).

Alongside a requirement for Integrins in mediating basal surface adhesion, signaling through Integrins has been shown to be required for apical-basal polarity in some epithelia and is known to influence cell–cell adhesion through adherens junctions [53]. Remodeling of these junctions underpins cell apical geometry remodeling. Examining the apical surface of *talin RNAi* and *Mys*^*DN*^ retinas revealed defects in cell apical geometry compared to wild-type retinas. These defects included elongated tertiary pigment cells, mispositioning of cells, and surnumerous bristle cell complexes. However, most retinal cells could still be identified based on their position and apical geometry within the ommatidium (Fig 3A–3C). Altogether, we conclude that Integrin adhesion is required for cell basal geometry remodeling during retinal morphogenesis. Our results also show that Integrins are required for patterning and cell geometry remodeling at the apical surface of the epithelium.

### Cell basal geometry remodeling begins with patterning of the ECM through localized Laminin accumulation

**Fig 4:**
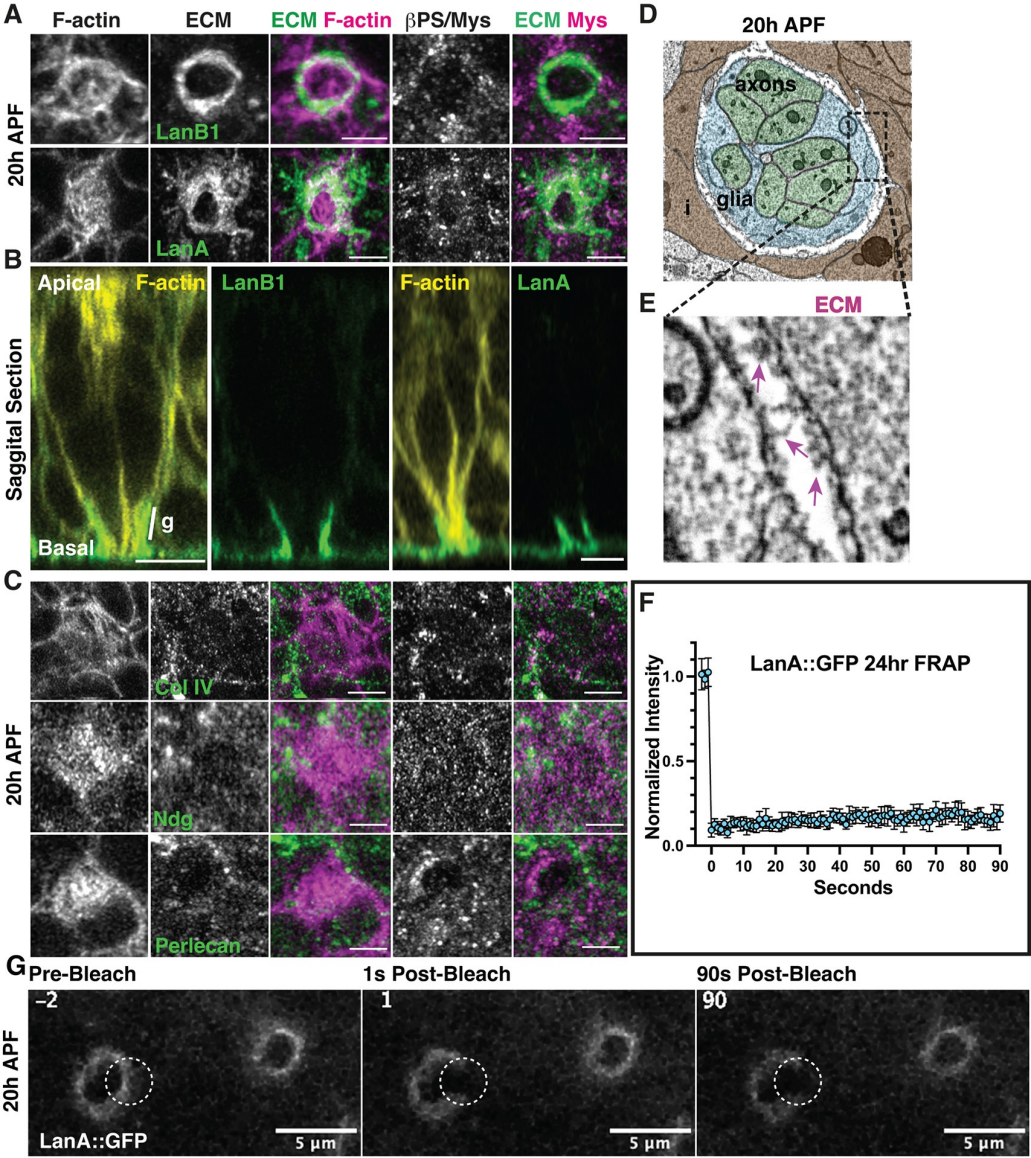
Laminin accumulation at the presumptive grommet precedes that of Integrins. (A-C) Basal surface of a retina imaged at 20h APF, before the onset of cell basal geometry remodeling. (A) The β-laminin subunit LanB1and LanA accumulate at the presumptive grommet. F-actin (magenta), LanA and LanB (green), βPS/Mys (magenta). Scale bars: 2 μm. (B) Sagittal section showing accumulation of LanB1and LanA at the presumptive grommet. F-actin (yellow), LanB1 and LanA (green). Scale bars: 5 μm. (C) Collagen IV, Ndg, and Perlecan show a punctate distribution at the basement membrane, with no enrichment around the grommet. F-actin (magenta), Col-IV, Ndg, Perlecan (green), Mys (magenta). Scale bars: 2 μm. (D, E) Electron micrographs of a presumptive grommet at 20h APF. (D) is a proximal section through one ommatidium, taken at the level of the photoreceptor axons (green). The region indicated by a black dashed box is shown at a higher magnification in (E). Pink arrows point to the electron dense extracellular matrix (ECM) lining the glial cell (blue) that surrounds the axon bundle, and the interommatidial cells (brown). (F, G) FRAP of LanA::GFP at 20h APF. Stills from a representative movie are shown in (G). Data associated with panel (F) can be found in S2 Data.

Basement membrane organization influences morphogenesis through both biochemical and mechanical regulation [54]. This prompted us to examine the relationship between the basement membrane, Integrin adhesion, and cell basal geometry remodeling. To this end, we examined the localization and requirement of the Laminin A and B1 subunits (Lamininα, LanA and Lamininβ, LanB1), Perlecan/Trol, Collagen-IV/Viking (Col-IV), the glycoprotein Nidogen (Entactin/Ndg), and the secreted glycoprotein protein-acidic-cysteine-rich (Sparc), which are all components of the basement membrane [2]. For Laminin, Ndg, and SPARC, we used strains generated from a fosmid library that express a functional GFP-tagged transgene under the control of their own respective promoters [55–57]. For Col-IV and Perlecan, we used functional GFP exon-trap strains [58]. We examined the retina of these animals and found that as early as 20h APF, 12 h before the αPS1/Mew-βPS/Mys Integrin receptor is polarized in the interommatidial cells, both the LanA and LanB1 chains accumulate at the center of the ommatidium in a pattern resembling the grommet structure (Figs 4A, 4B, S5A and S5C). At this developmental stage, Col-IV, Ndg, and Perlecan are not enriched in the same manner and instead are distributed at low levels across the basal surface of the retina (Figs 4C,S6A, S6C and S6E). These results show that Laminin accumulation at the presumptive grommet precedes Integrin accumulation at this location. This suggests that Laminin patterns at the basal surface of cells serve as positional cues to control Integrin localization.

Our finding that Laminin accumulates at the presumptive grommet, but Col-IV, Ndg, and Perlecan do not, raise the question as to whether Laminin assembles into an ECM at this location. To examine this, we used electron microscopy (Fig 4D and 4E) and FRAP (Fig 4F). Cross sections through the presumptive grommet at 20h APF revealed that electron dense material lines the photoreceptor axon bundle and interommatidial cells (Fig 4D and 4E). This is consistent with Laminin assembling into an ECM. For FRAP, we used LanA::GFP. We found very little recovery after photobleaching LanA::GFP in defined regions of interest (Fig 4F and 4G). We therefore conclude that at 20h APF, Laminin accumulates at the presumptive grommet, where it forms a stable ECM.

**Fig 5:**
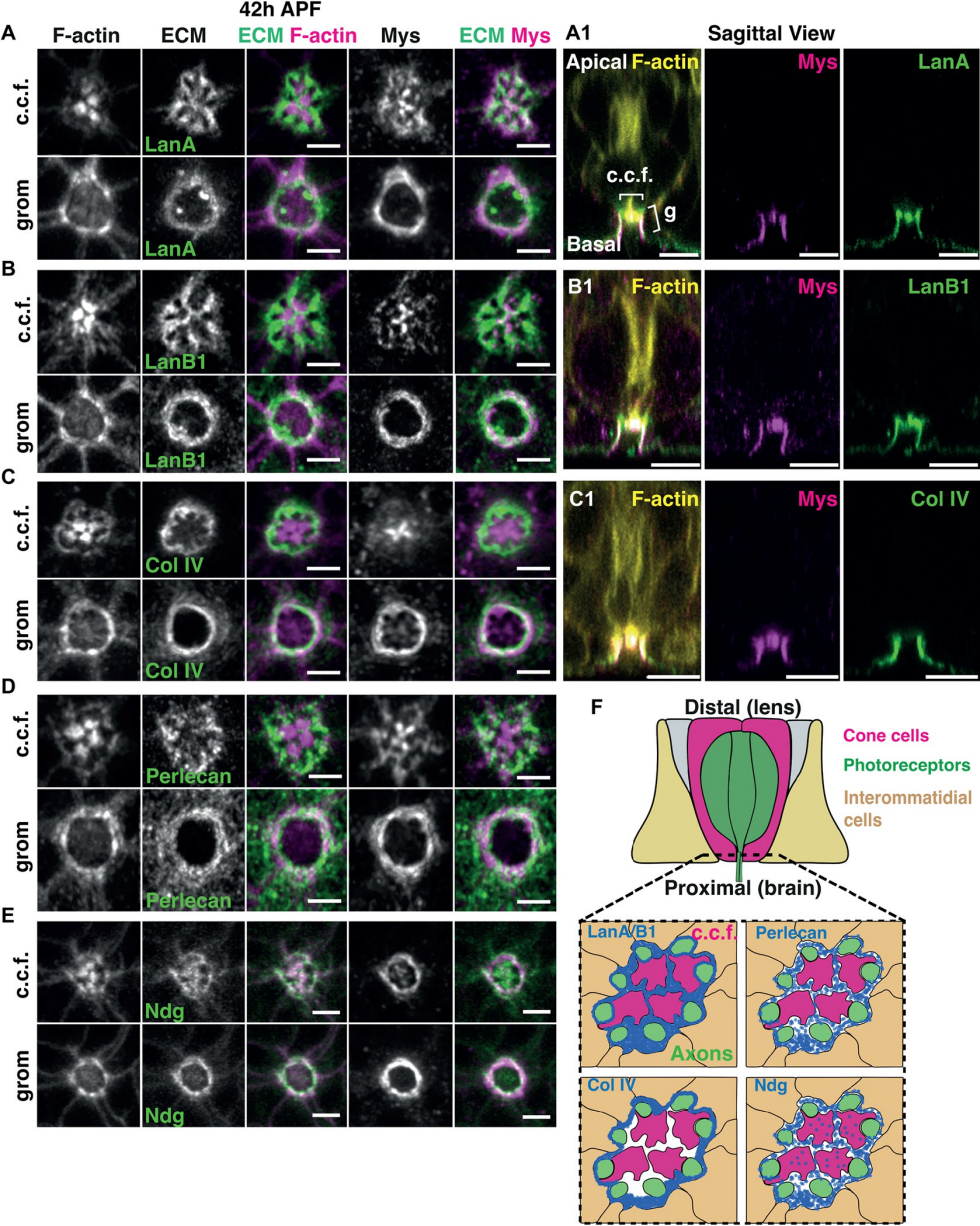
Laminin, Collagen-IV, Perlecan, and Ndg distribute differentially at the basal surface of the ommatidium. **(A-E)** Projections of basal sections around the feet of cone cells (c.c.f.) and at the grommet (grom, g), in 42h APF retinas, when cell basal geometry remodeling is completed. **(A, B)** Projection of 4 confocal sections for LanA **(A)** and LanB1 **(B),** encompassing the region where the feet of the cone cells contact the ECM, and projection of 4 confocal sections at a more proximal region, below the feet of the cone cells, showing the grommet structure encircling the photoreceptor axons. LanA/B (green) and F-actin (magenta). Distally, LanA/B1 localizes around the axons and in between the cone cell feet. Proximally, LanA/B localizes at the grommet. **(A1–B1)** Sagittal sections of a LanA::GFP and LanB1::GFP expressing retina. F-actin (yellow), βPS**/**Mys (magenta), and LanA/B1 (green). **(C)** Projection of 4 confocal sections for Collagen IV encompassing the region where the feet of the cone cells contact the ECM, and projection of 4 confocal sections at a more proximal region, below the feet of the cone cells, showing the grommet. Col-IV (green) and F-actin (magenta). Distally Col-IV localizes around the axon bundle but is not enriched in between the cone cell feet. Proximally, Col-IV localizes like LanA/B1, at the grommet. **(C1)** Sagittal section of a Col-IV::GFP retina. F-actin (yellow), βPS /Mys (magenta), and Col-IV (green). **(D)** Projection of 4 confocal sections for Perlecan encompassing the region where the feet of the cone cells contact the ECM, and projection of 4 confocal sections at a more proximal region including, below the feet of the cone cells, showing the grommet. Perlecan (green) and F-actin (magenta). Distally, Perlecan localizes in punctate structures, around the axons but not in between the cone cell feet. Proximally, Perlecan localizes at the grommet. **(E)** Projection of 4 confocal sections for Ndg, encompassing the region where the feet of the cone cells contact the ECM, and projection of 4 confocal sections at a more proximal region below the feet of the cone cells, and including the grommet. Distally, Ndg localizes below the cone cell feet. Proximally, Ndg localizes at the grommet. Sagittal sections of Perlecan::GFP and Ndg::GFP are provided in S6 Fig. **(F)** Drawing recapitulating the pattern of expression of LanA/B1, Col IV, Perlecan, and Ndg in the distal part of the ommatidium center. Scale bars **(A, B, C, and D, E)**: 2 μm; **(A1, B1, and C1):** 5 μm.

Next, we examined the distribution of ECM components at a later stage of retinal development, when βPS/Mys is recruited at the grommet. We found that LanA/B, Col-IV, Ndg, and Perlecan all become enriched in proximal sections of the grommet (Fig 5). These components, however, accumulate at different locations along the proximal-distal axis of the retina. In distal sections, LanA and LanB1 localize around the cone cell feet and around axons of the photoreceptors (Fig 5A and 5B). Col-IV localizes around the axons but not between the cone cell feet (Figs 5C and S6B). Perlecan and Nidogen both accumulate around the cone cell feet (Figs 5D, 5E, S6D, S6F, S6G and S6H). Sparc localizes in punctate structures within the photoreceptor soma and axon (S7 Fig). These patterns of expression for LamininA/B1, Col-IV, Perlecan, Ndg, and Sparc are summarized in Fig 5F. We conclude that the basement membrane of the retina is a patterned compartment, showing differential accumulation of ECM components across its surface.

### Laminin is required for the polarized accumulation of Integrins

**Fig 6:**
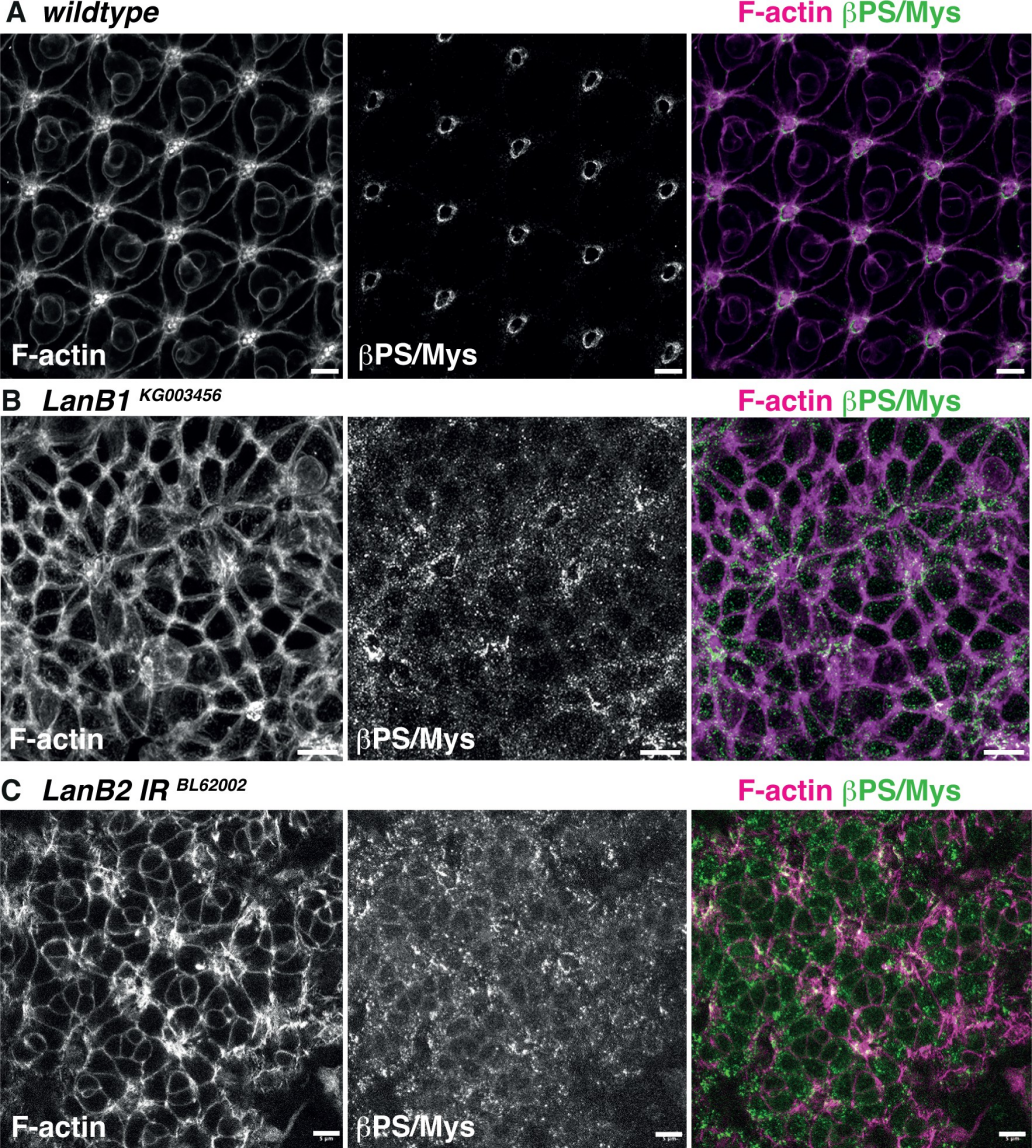
Laminin is required for cell basal geometry remodeling. **(A)** From top to bottom: Confocal images showing the apical and basal surface of a 42h APF wild-type retina F-actin (magenta) and βPS/Mys (green). **(B)** Confocal images showing the basal surface of a 42h APF retina within a mutant clone of the *LanB1*^*KG003456*^ allele. F-actin (magenta) and βPS/Mys (green). **(C)** Confocal images showing the basal surface of a 42h APF expressing a RNAi under the control of the GMR-Gal4, to target LanB2. F-actin (magenta) and βPS/Mys (green). Scale bars: 5 μm.

To test the idea that localized Laminin accumulation induces interommatidial cell basal geometry remodeling by recruiting Integrins, we sought to perturb the expression of this ECM component while monitoring βPS/Mys localization. For this, we used both a loss-of-function allele of *LanB1* and RNAi against *LanB2*. Consistent with our model, we found that in both cases, βPS/Mys Integrin localization was affected when compared to wild type (Fig 6A–6C). βPS/Mys failed to accumulate at the grommet and instead was distributed at the basal plasma membrane into punctate domains (Fig 6B and 6C). In addition, these perturbation experiments affected cell basal geometry remodeling. The strong effect of these perturbations on cell shape prevented us from assigning cell types from the apical surface and prevented us from analyzing the data by PCA.

Consistent with basement membrane regulation being important for cell basal geometry remodeling, we found that degrading the basement membrane by expressing Matrix Metalloproteases MMP1 or MMP2 in retinal cells leads to a failure in βPS/Mys localization at the grommet and prevents cell basal geometry remodeling (S8A–S8D Fig). In vitro studies have shown that recombinant *Drosophila* MMP1 can degrade Col-IV, but not Laminin, and that MMP2 can degrade both Col-IV and Laminin [59]. The MMP2 phenotype we observed in basal surface organization is stronger than that of the MMP1 overexpression. These results, therefore, suggest that both Col-IV and Laminin play a role in controlling the basal geometry of retinal cells. This suggestion is consistent with our finding that both these basement membrane proteins are enriched at the grommet once cells have acquired their basal geometry.

### The Dystroglycan complex is required for localized Laminin accumulation in basement membrane development

**Fig 7:**
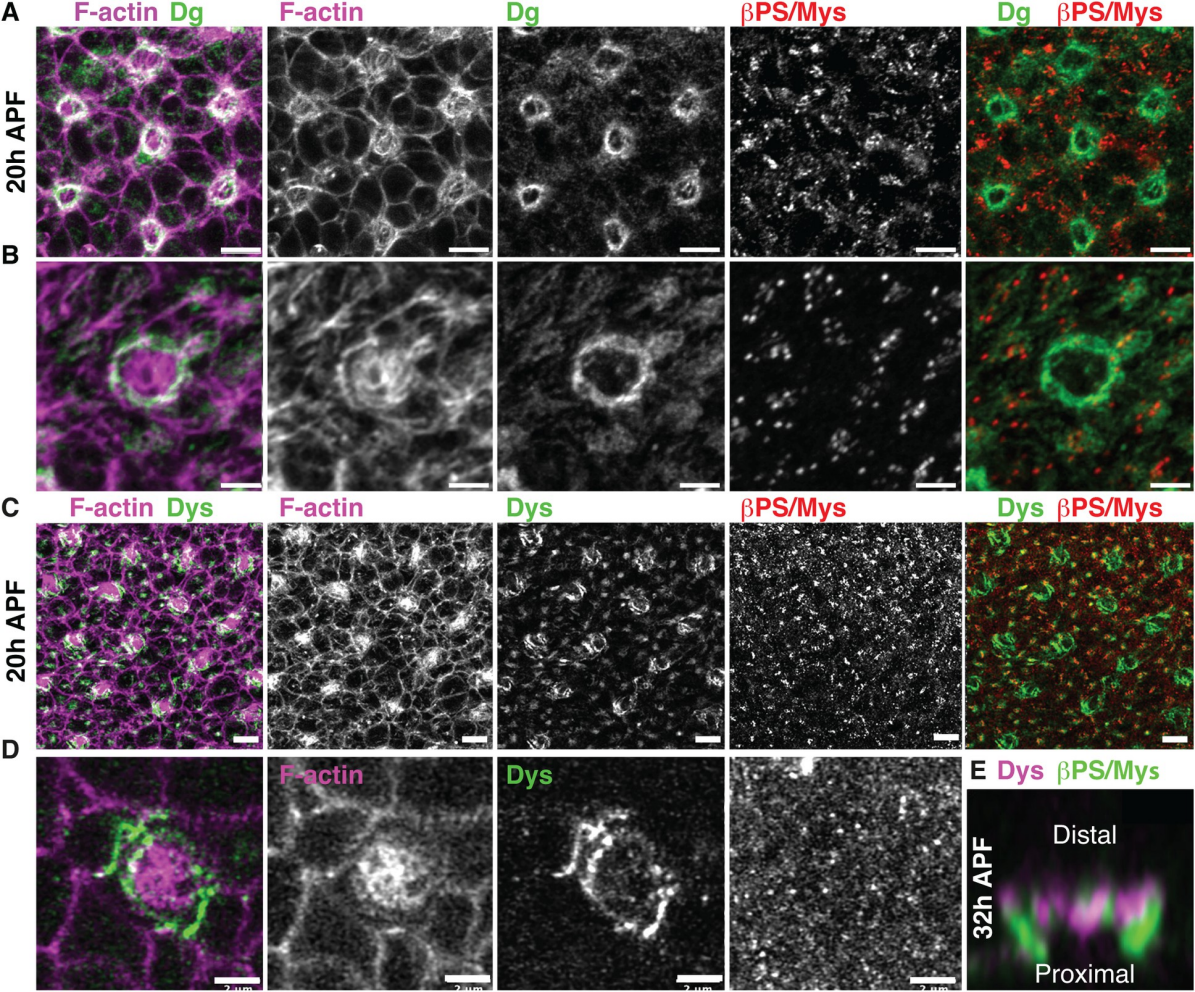
Dg and Dys accumulate at the presumptive grommet. **(A)** Confocal images showing a retinal basal surface at 20h APF and a close-up view **(B)** of the presumptive grommet for one ommatidium. F-actin (magenta), Dg::GFP (green), and βPS/Mys (red). **(C)** Confocal images showing a basal retinal surface at 20h APF and a close-up view **(D)** of the presumptive grommet for one ommatidium. F-actin (magenta), Dys::GFP (green), and βPS/Mys (red). **(E)** Sagittal section of a 32h APF grommet showing the relative distribution of βPS/Mys (green) and Dys::GFP (magenta**).** Scale bars: **(A, C)** 5 μm; **(B, D, E)** 2 μm.

Our experiments suggest an early role for Laminin in promoting the accumulation of αPS1/Mew-βPS/Mys Integrin receptor at the grommet. The DGC is required to organize Laminin in basement membrane maturation in several model systems [43,44,46,60]. These previous findings prompted us to examine the function of DGC components in localizing Laminin within the retinal basement membrane. Firstly, to examine the expression of Dg, we made use of endogenously tagged Dg::GFP [61] and the exon trap Dystrophin::GFP (Dys::GFP), which tags the long isoform of this protein [62]. We found that both Dg::GFP and Dys::GFP accumulated at the presumptive grommet at 20h APF (Fig 7A–7D), approximately 12 h before Integrins can be detected at this location. In addition, these proteins localize with Integrin clusters at the basal surface of retinal cells (Fig 7A and 7C). Examining the basal surface of the retina at 42h APF, as the basal geometry of cells has been remodeled, showed that Dys::GFP localizes at the grommet and also at the cone cell feet (S9 Fig). At these locations, Dys::GFP and βPS/Mys do not fully overlap, which is consistent with the DGC and Integrins assembling distinct adhesion sites (Fig 7E).

**Fig 8:**
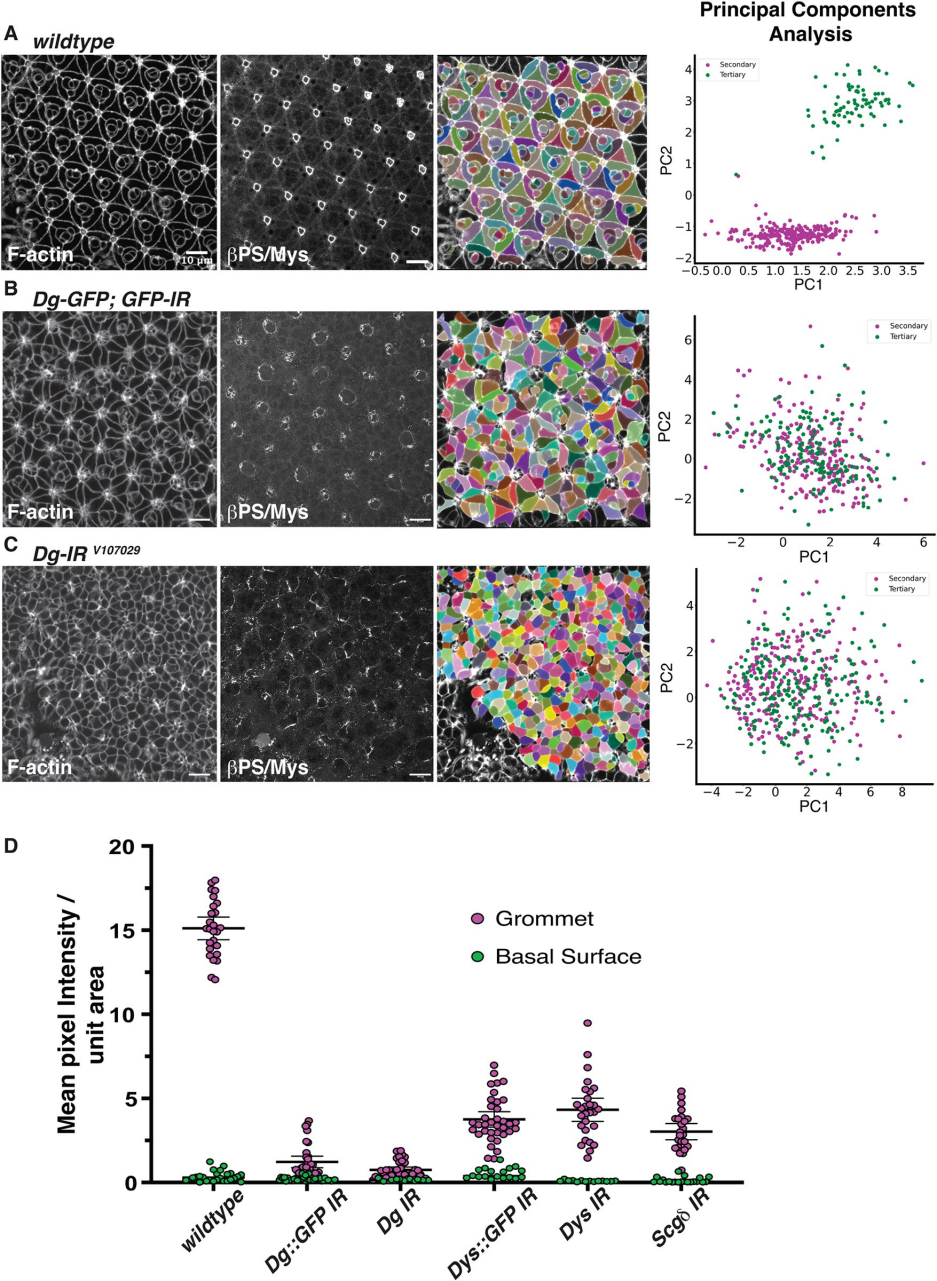
Dg is required for Integrin polarization in cell basal geometry remodeling. **(A)** From left to right: Confocal section of a wild-type patterned retinal basal surface at 42h APF stained for F-actin and βPS/Mys (integrins). Segmentation of the basal surface using the F-actin channel and napari. PCA distinguishing basal geometry of the secondary (magenta) and tertiary (green) pigment cells. **(B)** From left to right: Confocal section of a 42h APF retina expressing a GFP-tagged version of Dg and an RNAi to target GFP, expressed using the GMR-Gal4 driver. F-actin and βPS/Mys (integrins). Segmentation of the basal surface using the F-actin channel and napari. PCA distinguishing basal geometry of the secondary (magenta) and tertiary (green) pigment cells. **(C)** From left to right: Confocal section of a 42h APF retina expressing an RNAi under the control of GMR-Gal4, to target Dg. F-actin and βPS/Mys (integrins). Segmentation of the basal surface using the F-actin channel and napari. PCA distinguishing basal geometry of the secondary (magenta) and tertiary (green) pigment cells. **(D)** Quantification of the amount of Integrin (βPS/Mys) detected at the grommet and at the basal surface of wild-type 42h APF retinas, Dg-GFP expressing RNAi against GFP, retinas expressing Dg RNAi (IR), Dys-GFP expressing RNAi against GFP, retinas expressing Dys RNAi (IR), and retinas expressing Sarcoglycanδ (Scgδ) RNAi (IR). Scale bars: 10 μm. Data associated with panels **(A-C)** and panel **(D)** can be found in S3 and S4 Data, respectively.

In our model of cell basal geometry remodeling, the DGC patterns the basal tissue surface by generating Laminin-rich sites, and these sites direct Integrin adhesion. To test this, we generated *Dg*-deficient retinal tissue using the eyflp-FRT system and the *Dg*^*O86*^ allele, which encodes for a truncated Dg protein lacking the ECM-binding of the C-terminal αDg and β-Dg domains [63]. In these experiments, only few, sparse mutant cells could be recovered (S10 Fig). However, large wild-type twinspots were readily visible [64]. This suggests that *Dg* is required for cell proliferation or survival (S10 Fig). To gain further insight into *Dg* function in retinal morphogenesis, we used RNAi to decrease the expression of *Dg*. Firstly, we used RNAi against GFP to target endogenously tagged Dg (Fig 8). Examining the basal surface of the tissue, we found the basal geometry of Dg::GFP RNAi cells was affected compared to wild type (Fig 8A and 8B). PCA revealed that cell type–specific basal geometry was lost, as the secondary and tertiary pigment cells no longer clustered together (Fig 8B). We note here that we could still detect GFP signal at the grommet, indicating that the RNAi did not completely inhibit Dg expression (S11A Fig). Secondly, we made use of 2 RNAi lines that directly target independent regions of Dg. Expressing either of these RNAi interfered with basal cell geometry remodeling (Figs 8C and S12A–S12C). In addition, we observed a significant reduction in Integrin accumulation at the grommet compared to the wild type (Fig 8C and 8D).

**Fig 9:**
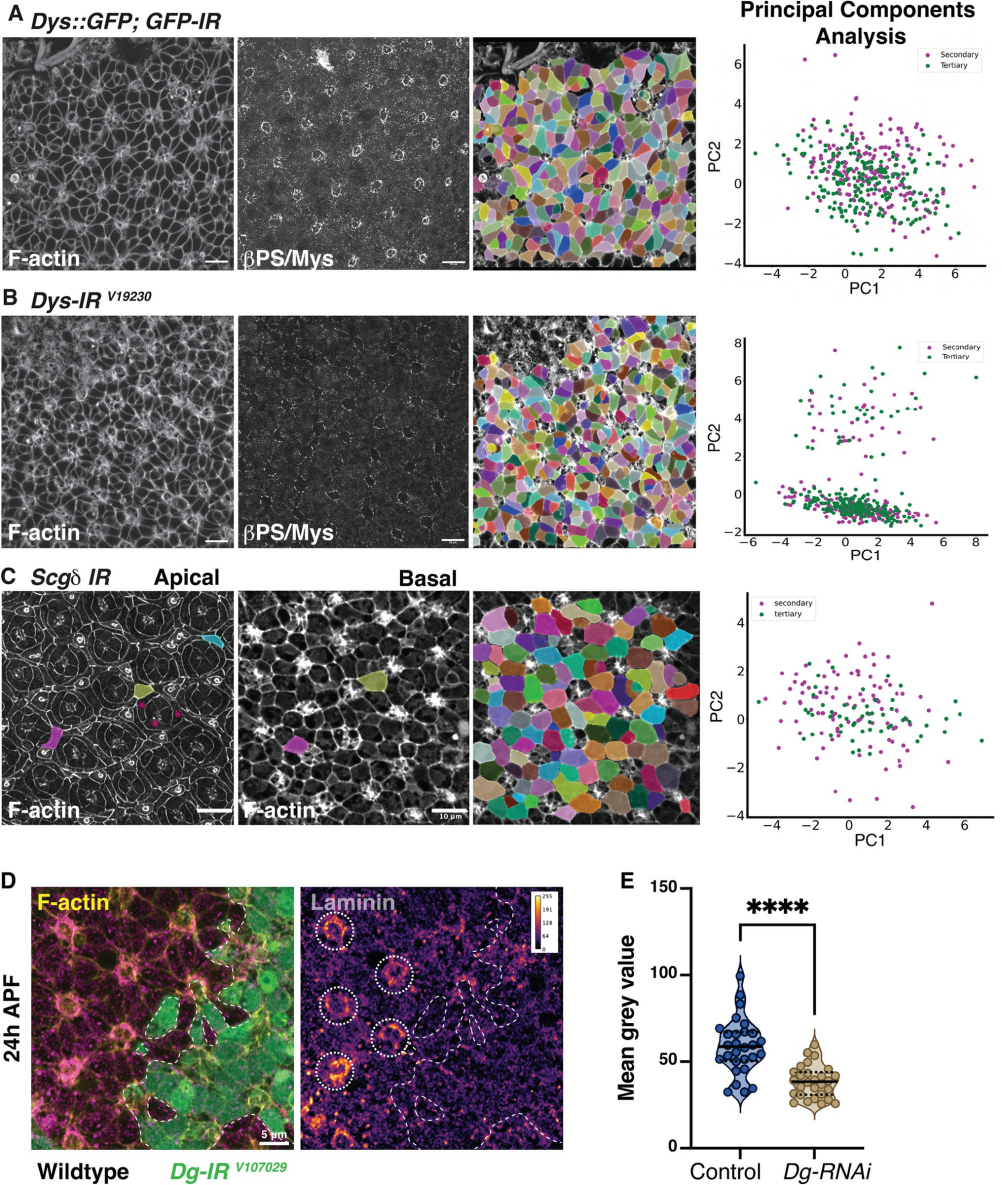
Dg in required for local Laminin accumulation and Integrin polarization. **(A)** From left to right: Confocal section of a 42h APF retina expressing a GFP-tagged version of Dys and an RNAi to target GFP, expressed using the GMR-Gal4 driver. F-actin and βPS/Mys (integrins). Segmentation of the basal surface using the F-actin channel and napari. PCA distinguishing basal geometry of the secondary (magenta) and tertiary (green) pigment cells. **(B)** From left to right: confocal section of a 42h APF retina expressing an RNAi under the control of GMR-Gal4, to target Dys. F-actin and βPS/Mys (integrins). Segmentation of the basal surface using the F-actin channel and napari. PCA distinguishing basal geometry of the secondary (magenta) and tertiary (green) pigment cells. **(C)** From left to right: confocal section of the apical surface and basal surface of a 42h APF retina expressing an RNAi under the control of GMR-Gal4, to target Scgδ. F-actin and βPS/Mys (Integrins). Segmentation of the basal surface using the F-actin channel and napari. PCA distinguishing basal geometry of the secondary (magenta) and tertiary (green) pigment cells. Scale bars in **A-C**: 10 μm. **(D)** Basal surface of a 24h APF retina expressing Dg-RNAi using the coinFLP system. Cells expressing RNAi are marked in green and outlined using a dashed line. Wild-type grommets are circled using a dashed line. Scale bar: 5 μm. **(E)** Quantification of the Laminin signal in the wild-type and Dg-RNAi. *P* = < 0.0001, using an unpaired *t* test with Welch’s correction.

In addition, we targeted Dys using the same methodology, inhibiting the expression of Dys::GFP using the GFP RNAi line (Fig 9A), and 2 RNAi lines targeting independent regions of the gene (Figs 9B and S12D–S12F). In all 3 cases, the genetic manipulation impaired basal geometry remodeling. In addition, these manipulations also led to a significant reduction of Integrin accumulation at the grommet (Fig 8D). As observed for Dg::GFP, we could still detect residual Dys::GFP at the grommet in the GFP RNAi experiment, indicating partial knockdown only (S11B Fig). Finally, we used RNAi to target *Sarcoglycanδ (Scgδ*,), which is a component of the DGC (Fig 9C). This led to a complete failure in basal geometry remodeling, consistent with this factor playing a role in the DGC pathway.

To better describe how our RNAi-based manipulations of the DGC affect basal cell shape, we performed PCA on pairwise combinations. We first compared the secondary and tertiary cells where GFP RNAi was used to inhibit expression of Dg::GFP and Dys::GFP (S13 Fig). Consistent with Dg and Dys functioning together as part of the DGC, we found the PCA analyses and correlation circle plots for the secondary and tertiary pigment cells to be remarkably similar for these 2 genotypes (S13A and S13B Fig). Thus, these genetic perturbations have similar effects on cell basal geometry. For the secondary pigment cells, inhibiting Dg::GFP and Dys::GFP led to an increase in cell roundness (S13C Fig). The geometry of the tertiary also became more variable (S13D Fig).

We then extended our PCA comparisons to include the following pairs: GFP RNAi against Dg::GFP and Dg RNAi (S14 Fig), GFP RNAi against Dys::GFP and Dys RNAi (S15 Fig), Dg RNAi and Dys RNAi (S16 Fig). For each pair, we first compared all interommatidial cells of each genotype to wild type. For all these genotype comparisons, the correlation circle plots differed from wild type in a similar manner (S14A–S14C, S15A–S15C and S16A–S16C Figs), indicating that they had similar effects on cell basal geometry. We then performed a comparison of either the secondary or tertiary cells of each paired genotype to each other and generated correlation circle plots to understand which parameters explain most of the variance in the PCA. In all comparisons, cells failed to form discrete clusters (S14D, S14E, S15D, S15E, S16D and S16E Figs). In all cases, cell roundness contributed to variability along the PC1 axis, with our RNAi manipulations leading to an increase in roundness of the secondary cells and more variability in the roundness of the tertiary cells (S14F, S14G, S15F, S15G, S16F and S16G Figs).

Finally, we compared the Scgδ and Dg RNAi conditions (S17 Fig). We found that these phenotypes differed from wild type in a similar manner (S14A–S14C Fig). When comparing either the secondary or tertiary cells of each paired genotype, there was significant overlap between the cell populations (S17D and S17E Fig). In this comparison, Scgδ RNAi led to an increase in circularity of both the secondary and tertiary cells when compared to wild type (S17F and S17G Fig). Altogether, this part of our work indicates that the DGC is required for polarized accumulation of Integrin at the basal surface of retinal cells during basal geometry remodeling.

Our quantification of the *Dg*, *Dys*, and *Scgδ* RNAi phenotypes, together with the early accumulation of both Laminin and Dg-Dys at the presumptive grommet, suggest that Laminin and DGC function in cell basal geometry remodeling are linked and that the DGC might promote the accumulation of Laminin in the retinal ECM. To test this idea, we inhibited the expression of DGC using RNAi against Dg and *Scgδ* and asked whether this affected early Laminin accumulation at the presumptive grommet. Both these knockdowns led to a decrease in Laminin accumulation at the presumptive grommet (Figs 9D, 9E and S18). These results show that the DGC is required for patterning the developing retinal basement membrane by promoting local Laminin accumulation.

## Discussion

We have used the *Drosophila* retina as a model system to study the pathways that regulate remodeling of a tissue basal surface during epithelial morphogenesis. In retinal development, cells remodel their basal geometry, and our work shows that this remodeling begins with establishing a pattern of Laminin-rich domains, distributed across the developing basement membrane. We find that establishing this Laminin pattern depends upon the DGC. Once established, this pattern of Laminin sites directs the recruitment of Integrins, and this recruitment coordinates basal geometry remodeling across groups of cells. Thus, we propose that patterning of a basement membrane through local Laminin accumulation can determine the basal polygonal geometry of epithelial cells by controlling the localization of Integrin adhesion. In the retina, failure to establish specific cell basal geometries will compromise eye function, as cell shape and function are intimately linked in this sensory epithelium.

### The DGC promotes localized Laminin accumulation to pattern the retinal ECM

Dg is required for basement membrane formation in several tissues. For example, Dg knockout mice fail to develop beyond 5.5 embryonic days due to a failure in assembling the extraembryonic basement membrane, called the Reichert’s membrane [60]. Examining Dg function during mouse embryoid body formation revealed that this cell surface receptor is required to organize Laminin-1 into fibrils [46]. The authors proposed that Dg and Integrins cooperate to rearrange Laminin-1 into specific structures—fibrils and plaques [65]. Dg can also potentiate Integrin adhesion to Laminin in Caco-2 intestinal epithelial cells [66]. Interestingly, when Dg is overexpressed in the developing fly trachea, it leads to precocious Laminin accumulation in the ECM lining these epithelial cells [47]. Dg is also required for organizing Laminin at the basal surface of the fly follicular epithelium [43]. Altogether, these studies suggest that basement membrane assembly depends upon the DGC and Integrin, which might play different, yet overlapping roles in the BM assembly process. Our results in the retinal epithelium show that the DGC is required in the interommatidial cells to organize their basal ECM. Based on our inhibition of *Dg* and *Scgδ*, which led to a reduction in Laminin accumulation at the presumptive grommet, we propose that the DGC is required to promote Laminin accumulation at this location. We envisage that this is achieved through DGC binding to Laminin. Alternatively, it is possible that the DGC regulates the expression or secretion of Laminin. Further work will be required to distinguish between these possibilities.

We note that a previous study showed that early in retinal development, Dg localizes at the apical membrane of the photoreceptors. This study proposed a role for Dg in promoting elongation of these sensory neurons, independently to any potential role this surface receptor might play in basement membrane organization [67]. This conclusion was based on *Df(2R)Dg*^*248*^ mutant clones and *trans*-heterozygous retinas, where Dg function was impaired in all retinal cell types. Moreover, the basement membrane was not examined in this study. Our work, making use of multiple, independent RNAi lines to inhibit the expression of Dg and Dys, adds to this function by showing that the Dg receptor and its interactor Dys are required to organize the basement membrane and the basal cell shape of retinal cells. We find that Laminin and the DGC accumulate at the presumptive grommet before retinal cells undergo basal geometry remodeling. Electron microscopy at this early stage of retinal development shows that Laminin is part of an ECM around the photoreceptor axons and an ECM that lines the feet of the interommatidial cells that surrounds them. We do observe a space between these 2 ECMs, which might be an artifact of sample preparation—perhaps a weak spot that tends to rupture upon dissection and fixation of the sample. Alternatively, it is possible that these are 2 independent Laminin deposits, separated by a gap of interstitial milieu. Nevertheless, our RNAi approach using the eye-specific GMR-Gal4 driver line indicates that Laminin, Dg, and Dys are produced by retinal cells. Whether all retinal cells express these components or different cell types express either Laminin and/or Dg remains to be tested.

The idea that different cell types in a tissue can express different ECM components to induce patterning of a basement membrane is well supported by recent work in the mouse hair follicle. In this sensory organ, the architecture and composition of the basement membrane is highly specialized depending on the cell–cell and cell–tissue interface considered [68–70]. Moreover, different cell populations, including epithelial stem cells and fibroblasts, express different ECM components in the hair follicle [71], supporting the notion that specific basement membrane organization contributes to cell–cell communication and overall 3D tissue architecture. Our time course experiments indicate that Col-IV, Perlecan, and Ndg are distributed across the basal surface of the retina. These factors incorporate into the grommet after Laminin and Dg. Such a temporal sequence, whereby Laminin is localized before Collagen-IV is also seen in the fly embryo [72] and in the developing trachea [47]. Therefore, in various instances of basement membrane assembly, Laminin deposition appears to precede that of Collagen-IV.

### Controlling Integrin adhesion contributes to determining the basal geometry of cells

What role could a Laminin ECM play in tissue morphogenesis? Part of the answer to this question may be found when examining the relationship between the DGC, Laminin, and Integrins in basement membrane formation in the developing *Drosophila* oocyte [43,44,73]. In this tissue, Dg is required to generate Laminin fibrils that line the basal surface of the follicular epithelium, which surrounds the oocyte. In addition, Dg promotes formation of basal F-actin fibers in the follicular cells, which align with the Laminin fibrils. In addition, Integrin adhesion is required to organize the basal F-actin cytoskeleton, and cells deficient in Integrins present a reduced basal surface area when compared to their wild-type counterparts [42,74]. In this tissue, alignment of Laminin fibrils and basal F-actin is thought to generate a corset, which mechanically constrains the oocyte to control its axis of elongation. Thus, a combination of the DGC, Laminin, and Integrin adhesion regulates basal surface area of the follicular epithelial cells and the overall shape of the egg. In the fish optic cup, βPS Integrin and Laminin C1 are required for cell basal contraction, which promotes tissue curvature, essential for generating a cup-like structure [11,14,75]. In all these epithelia, Integrins organize the basal actomyosin cytoskeleton and provide anchoring points against which this contractile machinery can pull to generate traction.

Integrins have also been implicated in cell basal contraction in the *Drosophila* retina; however, earlier work using a thermosensitive loss-of-function allele of βPS/Mys had ruled out a role for this adhesion receptor in cell basal geometry remodeling [48]. Our work using both *talin* RNAi and the strong *mys*^*1*^ loss-of-function allele [76] shows a clear requirement for Integrin adhesion in cell basal geometry remodeling. We found that prior to cell basal geometry remodeling, retinal cells present 1 cluster enriched in βPS/Mys Integrin. This type of organization for Integrin adhesion sites were previously missed [48]. As the interommatidial cells remodel their basal geometry, the Integrin clusters is no longer detected. Integrin localization becomes polarized such that this surface receptor concentrates at the grommet. Therefore, cell basal geometry remodeling correlates with a switch in Integrin localization, from a cluster to polarized in the plane of the basal retinal surface. We propose that localized accumulation of the DGC and Laminin can direct Integrin polarization to coordinate cell shape remodeling in epithelial morphogenesis. Our experiments show that MMP2 overexpression leads to a stronger phenotype than MMP1. In vitro, MMP2 can catalyze proteolysis of both Col-IV and Laminin, while MMP1 has been shown to catalyze proteolysis of only Col-IV [59]. Therefore, our results suggest that in addition to Laminin, Col-IV is also required for cell basal geometry remodeling.

We also note that, as well as regulating cell basal geometry remodeling in the retina, our results indicate that interfering with Integrins and DGC expression leads to defects in cell positioning, number, and apical geometry remodeling. It will be interesting to establish how these basal surface receptors regulate apical surface morphogenesis.

### Cone cells express 2 Integrin receptors, αPS1/Mew-βPS/Mys and αPS2/if-βPS/Mys

We found that while the interommatidial cells express αPS1/Mew-βPS/Mys, the cone cells express both αPS1/Mew-βPS/Mys and αPS2/if-βPS/Mys. Thus, different cell types express different αPS subunits. It is not clear why the cone cells express 2 α-subunits. In the developing follicular epithelium of the fly oocyte, cells switch from expressing αPS1/Mew-βPS/Mys, to expressing αPS2/if-βPS/Mys [41]. In this tissue, the developmental switch between αPS1 and αPS2 expression was shown to correlate with a change in stress fiber orientation. In addition, αPS1-βPS/Mys was shown to be required to control F-actin levels basally. *αPS1* mutant cells presented elevated levels of F-actin, a phenotype not seen in *αPS2* mutant cells. Remarkably, in this tissue, *αPS2-*βPS/Mys, but not αPS1/Mew-βPS/Mys, was able to recruit the integrin adapter Tensin. The authors envisaged that the *αPS2* Tensin interaction might confer robustness in basal surface remodeling. With analogy to the follicular epithelium, we speculate that in the cone cells, *αPS1-*βPS/Mys and αPS2/Mew-βPS/Mys synergize in mediating robust attachment to the basement membrane to ensure these cells do not detach as the retina lengthens along the apical-basal axis [48].

## Material and methods

### Fly strains and genetics

Flies were raised on standard food. The following fly strains were used:

*Ubi-Ecadherin*::*GFP* [77]

*vkg*::*GFP (BL98343)* [58]

*LanA*::*GFP (v318155)*, *LanB1*::*GFP (v318180)*, *Ndg*::*GFP (v318629) and SPARC*::*GFP (v318015)* [55]

*Ubi-Myspheroid*::*GFP* [78]

*UAS-Torso*::*Mys*^*DN*^ [50]

Dystroglycan::GFP [61]

*Dystrophin*::*GFP* (BL59782) [62]

*Mew-YFP (Kyoto 115524) if-YFP (Kyoto 115467) and perlecan*::*GFP (Kyoto 110836)* [79]

*GMR- Gal4* [51]. This Gal4 driver is expressed in all retinal cells, in the wake of the morphogenetic furrow, before the animal enters pupation. It remains expressed in retinal cells throughout pupal development.

*UAS-talin RNAi VDRC 40339* and *BL 33913*, *UAS-LanB1 RNAi VDRC 23119* [80], *UAS-Dys RNAi BL55641* [81] and *V19230* [44]; *Mys*^*1*^ *FRT19A/Fm7C (BL23862)*, *UAS-Dg RNAi BL34895 and V107029* [61], *UAS-Sarcoglycanδ RNAi BL55325*, *UAS-LanB2 RNAi BL62002* [82,83]; *UAS-Mmp1 BL58700* and *UAS-Mmp2 BL58704* [84]; *UAS-GFP RNAi BL9330 and BL9331; FRT42D*, *Dg*^*O86*^ *BL63049* [63].

RNAi and Mmp1/2 experiments were performed at a range of temperatures, from 25°C to 29°C, to modulate the strength of expression through the Gal4/UAS system [85].

### Antibody staining

Retinas of appropriately staged animals were dissected in PBS on ice and fixed in 4% paraformaldehyde for 20 min at room temperature (RT) [86]. Retinas were washed in PBS-Triton 0.3% (PBS-T) then stained with primary antibody in PBS-T for 2 h at RT or overnight at 4°C. Retinas were washed in PBS-T and then stained with secondary antibodies for 2 h at RT or overnight at 4°C. Retinas were mounted in Vectashield (Vectorlabs). The following antibodies were used: rat DCAD2 anti-ECadherin (1:50), deposited to the DSHB by T. Uemura (DSHB Hybridoma Product DCAD2) [87], rabbit PA1-16730 anti-Laminin (1:20, Invitrogen), mouse CF.6G11 anti-Mys (1:20), deposited to the DSHB by D. Brower [88], combined with mouse or rat secondary antibodies conjugated to Dy405, Alexa488, Cy3, or Alexa647 (Jackson ImmunoResearch) as appropriate, used at 1:200, and phalloidin-TRITC (Sigma), to visualize F-actin. A minimum of 4 different retinas were imaged for each genotype.

### Confocal imaging and image processing

Images of fixed retinas were acquired on a Zeiss 900 confocal microscope with Airyscan2. Airyscan images were processed using the default settings offered by Zeiss. All images presented were processed using FIJI [89] and AdobePhotoshop CS4 (Adobe). Graphs were produced in GraphPadPrism 7 (GraphPad) or Python (seaborn and matplotlib). Figures were mounted in Adobe Illustrator CS4 (Adobe). In preparation for segmentation, Fiji was used to improve the cell membrane fluorescence signal with background subtraction (Rolling ball radius 50 pixels) and by applying a 3D Gaussian blur filter (Sigma 1.5). 3D segmentation was performed on samples stained with Ecadherin and phalloidin to segment cell membranes. Using Imaris 9.1.2, cells were manually segmented. 2D contours were drawn around each cell every 2 Z slices, and surfaces were created to render individual cells in 3D.

Quantification of Integrins at the grommet and at the basal surface was performed using Fiji. ROIs were manually defined using the hand drawing tool. To measure integrin fluorescence intensity at the grommet, ROIs were drawn around the outer and inner periphery of the grommet region. For integrin measurements at the basal surface, ROIs were drawn at random locations in confocal sections more proximal to the grommet. Grommet and basal surface mean pixel intensity values were then divided by the respective area to express the mean pixel intensity per area unit for each measurement.

### FRAP experiments

LanA::GFP and GMR-Gal4, UAS-Secreted-GFP white pupae were selected and incubated at 25°C for 20 h. Animals were prepared for imaging by manually removing a portion of the pupal case covering the head of the animal and then positioned on a cushion of BluTac that was fixed in place on a glass slide with double-sided tape. A coverslip containing a small drop of Voltalef oil was placed in contact with 1 retina [90]. *n* = 11 Lan::GFP FRAP experiments from 5 retinas were performed using a Leica DIVE microscope, and the results were analyzed using easy-FRAP and PRISM as previously described [91].

### 2D segmentation

Segmentation was performed on samples stained with phalloidin and Ecadherin to segment cell membranes and to assign cell types, respectively. Airyscan confocal images were processed with default Zeiss Airyscan processing parameters. Images were then processed with Napari [52] using the background subtraction filter (Rolling ball radius 50 pixels) and a Gaussian blur filter (Sigma/Radius 1.5) to enhance cell membrane signal for 2D segmentation. Segmentation, manual correction, and quantification were performed using Napari. Cells were automatically segmented in 2D using the Cellpose plugin with the cyto1 model. The masks generated from the automatic segmentation were then manually corrected in Napari. Cell parameters were calculated from the segmented masks using regionprops from scikit-image. All cell types, including the secondary and tertiary pigment cells, were identified based on their apical geometry and positioning within the ommatidia. If the cell type could not be identified based on its apical geometry, this cell was removed from the analysis.

### Principal component analysis

PCA was carried out using the Scikit-learn library in Python. The Standard scaler package was used to standardize the data across all metrics before calculating the principal components. The PCA package was then used to perform the PCA. Metrics included in the PCA were as follows: extent, major axis length, minor axis length, eccentricity, roundness, circularity, area, cell shape index, and perimeter.

The cell types, secondary and tertiary pigment cells, were assigned by following the cells in 3D to the apical surface where the cell types could be identified. Cells that could not be clearly assigned as either secondary or tertiary pigment cells were excluded from the PCA.

**Extent** is the area of an object divided by the area or the smallest rectangle (bounding box) that can fit around the object.


$$Extent=\frac{object\;area}{bounding\;box\;area}$$

**Major axis length** is the longest line that can be drawn through an object.

**Minor axis length** is the line that can be drawn through an object which is perpendicular to the major axis.

**Eccentricity** is the ratio of the length of the short (minor) axis to the length of the long (major) axis.


$$Eccentricity=\frac{length(minor\;axis)}{length(major\;axis)}$$

**Roundness** is a comparison of an object to the best fit circle of an object. The closer the object is to a perfect circle, the more round it will be.


$$Roundness=\frac{4(area)}{\pi {\left(major\;axis\right)}^{2}}$$

**Circularity** is a measure of the smoothness of an object.


$$Circularity=\frac{4\pi (area)}{{\left(convex\;perimeter\right)}^{2}}$$

**Cell shape index** is a dimensionless parameter to describe cell shape. When cells have smaller contacts with their neighbors the cell shape index is small.


$$cell\;shape\;index=\frac{perimeter}{\sqrt{area}}$$

Correlation circle plots were generated using the mlxtend plotting package in python using the plot PCA correlation graph function. PCA was performed on at least 3 retinas per genotype. Archived code generated to perform all PCA in this study can be found at https://doi.org/10.5281/zenodo.12942865

### Electron microscopy

Retinas were prepared for electron microscopy as in [92] but embedded in Epon 812 resin. Serial ultrathin sections were collected on ITO coated coverslips and imaged using a Sense backscattered electron detector in a Gemini 300 SEM with Atlas 5 for array tomography acquisition (Zeiss)—operating at 4.5 kV accelerating voltage, with 3 kV stage bias.

Data associated with panel **(E)** can be found in S5 Data.

## Supporting information

S1 Movie 3D rendering of a 20h APF ommatidium obtained after airyscan confocal imaging. (MP4)

S2 Movie 3D rendering of a 20h APF ommatidium obtained after airyscan confocal imaging. (MP4)

S3 Movie 3D rendering of a 32h APF ommatidium obtained after airyscan confocal imaging. (MP4)

S4 Movie 3D rendering of a 32h APF ommatidium obtained after airyscan confocal imaging. (MP4)

S1 Fig Retinal basal surface organization. F-actin staining revealing cell basal outlines in a 42h APF retina. Scale bar: 75 μm.
(EPS)

S2 Fig Integrin attachment is polarized in the interommatidial cells. **(A)** 42h APF secondary pigment cell expressing *talin* RNAi (magenta), surrounded by wild-type cells. βPS/Mys (green). Mosaic expression of *talin* RNAi is induced using the coin-FLP system [93]. In the close-up view, an arrow points to the lack of βPS/Mys corresponding to where the *talin*-deficient cell should contribute Integrins to the grommet. **(B)** 42h APF tertiary pigment cell expressing *talin RNAi* (magenta), surrounded by wild-type cells. GFP (magenta) marks the cell expressing the RNAi. In the close-up view an arrow, points to the lack of βPS/Mys at the location where the cell joins the grommet.
(EPS)

S3 Fig The interommatidial and cone cells express αPS1/Mew. **(A)** Basal surface of a 42h APF retina stained for αPS1/Mew::GFP (green) and F-actin (magenta). Cone cell feet (c.c.f.). The higher magnification shows the αPS1/Mew::GFP localization pattern at the grommet.
(EPS)

S4 Fig PCA and correlation circle plots. **(A-C)** Correlation circle plots for wild-type, *talin* RNAi, and *Mys*^*DN*^ retinas for both secondary and tertiary pigment cells. **(D)** PCA of secondary and **(E)** tertiary pigment cells comparing *talin* RNAi and *Mys*^*DN*^ retinas. Correlation circle plots to analyze the correlation between each variable and the principal components and how well the variables explain the variance of the data (bottom panels). **(F, G)** Parameter comparisons extracted from the original data. The 2 parameters that contribute most to principal component 1 are analyzed. Data associated with panels **(A-G)** are in S1 Data.
(EPS)

S5 Fig Laminin localization at the presumptive grommet precedes that of Integrins. **(A, B)** Basal surface of a 20h APF and 42h APF retina showing LanA::GFP accumulation at the grommet. **(C, D)** Basal surface of a 20h APF and 42h APF retina showing LanB1::GFP accumulation at the grommet. F-actin (magenta), LanA/B1 (green), Mys (magenta). Scale bars: 5 μm.
(EPS)

S6 Fig Localization of ECM factors in basal surface morphogenesis. **(A-F)** Projection of a few contiguous basal sections of retinas at 24h and 42h APF **(A, B)** Col-IV::GFP (green), F-actin (magenta), Mys (magenta). **(C, D)** Perlecan::GFP (green), F-actin (magenta), βPS/Mys (magenta). **(E, F)** Ndg::GFP (green), F-actin (magenta), βPS/Mys (magenta). **(G, H)** Sagittal sections of a Perlecan::GFP retina **(G)** and Ndg::GFP retina (**H**) at 42h APF. F-actin (yellow), βPS/Mys (magenta), and Perlecan/Ndg (green). Scale bars: 5 μm.
(EPS)

S7 Fig SPARC localizes in the photoreceptor cytosol and axon. **(A)** Projection of contiguous confocal sections taken in the plane of the photoreceptors’ cell body in a 24h APF retina. **(B)** Projection of contiguous confocal sections at the basal surface of a 42h APF retina. **(C)** Close-up view on photoreceptors soma in a 42h APF retina. **(D)** Close-up view of the basal surface of a 42h APF retina showing the photoreceptor’s axon bundles running across the tissue surface. F-actin (magenta), SPARC (green), and βPS/Mys (magenta). Scale bars: 5 μm.
(EPS)

S8 Fig Degrading the ECM by expressing MMPs prevents basal geometry remodeling. **(A)** Retina expressing MMP1 and **(C)** MMP2 under the control of the GMR-Gal4 driver, and close-up views of the center of an ommatidium, where the grommet is located, for retina expressing MMP1 **(B)** and MMP2 **(D)**. F-actin (magenta) and βPS/Mys (green).
(EPS)

S9 Fig Dys localizes at the grommet. **(A, B)** Basal retinal surface at 42h APF and a close-up view **(B)** of a grommet. F-actin (magenta), Dys::GFP (green), βPS (red). Scale bar **(A)**: 5 μm, **(B)**: 2 μm.
(EPS)

S10 Fig Dg is required for retinal cell proliferation or survival. **(A)** Confocal projection of a 42h APF retina of the following genotype: *eyFLP; ubiGFP*, *FRT42D / Dg*^*O86*^, *FRT42D;* An OrangeHot lookup table was used to distinguish *Dg*-mutant cells (lacking GFP) from twinspots (expressing 2 copies of GFP) and unrecombined heterozygous tissue (expressing 1 copy of GFP). One area of contiguous twinspot tissue is outlined with a solid blue line, while several much smaller *Dg*^*O86*^ clones are outlined by blue dashed lines and also indicated with a blue arrow. Scale bar = 50 μm.
(EPS)

S11 Fig GFP RNAi against Dg-GFP and Dys-GFP lead to partial knockdowns. **(A)** Confocal section showing the basal surface of a 42h APF retina expressing Dg::GFP and the RNAi targeting GFP. Note the residual GFP signal localized to a subset of grommets (white arrows). **(B)** Confocal section showing the basal surface of a 42h APF retina expressing Dys::GFP and the RNAi targeting GFP. Note the residual GFP signal localized at the grommets (white arrows). Scale bars: 10 μm.
(EPS)

S12 Fig The Dg-Dystrophin pathway is required for polarized accumulation of Integrins. **(A)** Apical and basal surface of a 42h APF retina expressing *Dg* RNAi under the control of the GMR-Gal4 driver. The basal surface has been segmented and cell basal geometry analyzed using PCA. **(B)** Basal surface of a 42h APF retina expressing *Dg* RNAi under the control of the GMR-Gal4 driver F-actin (magenta), βPS/Mys (green). **(C)** Close-up view of a grommet. **(D)** Apical and basal surface of a 42h APF retina expressing *Dys* RNAi under the control of the GMR-Gal4 driver. The basal surface has been segmented and cell basal geometry analyzed using PCA. **(E)** Basal surface of a 42h APF retina expressing *Dys* RNAi under the control of the GMR-Gal4 driver F-actin (magenta), βPS/Mys (green). **(F)** Close-up view of a grommet. Scale bars **(A, D)**: 10 μm; **(B, E)**: 5 μm; **(C, F)**: 5 μm. Data associated with panels **(A, D)** can be found in S1 Data.
(EPS)

S13 Fig Comparing Dg::GFP; GFP RNAi and Dys::GFP; GFP RNAi. **(A)** PCA of secondary and **(B)** tertiary pigment cells comparing *GFP RNAi* against *Dys*::*GFP* and *GFP RNAi* against *Dg*::*GFP* retinas. Correlation circle plots to analyze the correlation between each variable and the principal components and how well the variables explain the variance of the data (bottom panels). **(C, D)** Parameter comparisons extracted from the original data. The 2 parameters that contribute most to principal component 1 are analyzed. Data associated with **(A-D)** are in S3 Data.
(EPS)

S14 Fig Comparing Dg RNAi and Dg::GFP; GFP RNAi. **(A-C)** Correlation circle plots for wild type, *Dg RNAi*, and *GFP RNAi* against *Dg*::*GFP* retinas for both secondary and tertiary pigment cells. **(D)** PCA of secondary and **(E)** tertiary pigment cells comparing *Dg RNAi* and *GFP RNAi against Dg*::*GFP* retinas. Correlation circle plots to analyze the correlation between each variable and the principal components and how well the variables explain the variance of the data (bottom panels). **(F, G)** Parameter comparisons extracted from the original data. The 2 parameters that contribute most to principal component 1 are analyzed. Data associated with **(A-G)** are in S3 Data.
(EPS)

S15 Fig Comparing Dys RNAi and Dys::GFP; GFP RNAi. **(A-C)** Correlation circle plots for wild type, *Dys RNAi*, and *GFP RNAi* against *Dys*::*GFP* retinas for both secondary and tertiary pigment cells. **(D)** PCA of secondary and **(E)** tertiary pigment cells comparing *Dys RNAi* and *GFP RNAi* against *Dys*::*GFP* retinas. Correlation circle plots to analyze the correlation between each variable and the principal components and how well the variables explain the variance of the data (bottom panels). **(F, G)** Parameter comparisons extracted from the original data. The 2 parameters that contribute most to principal component 1 are analyzed. Data associated with **(A-G)** are in S3 Data.
(EPS)

S16 Fig Comparing Dys RNAi and Dg RNAi. **(A-C)** Correlation circle plots for wild type, *Dys RNAi*, and *Dg RNAi* retinas for both secondary and tertiary pigment cells. **(D)** PCA of secondary and **(E)** tertiary pigment cells comparing *Dys RNAi* and *Dg RNAi* retinas. Correlation circle plots to analyze the correlation between each variable and the principal components and how well the variables explain the variance of the data (bottom panels). **(F, G)** Parameter comparisons extracted from the original data. The 2 parameters that contribute most to principal component 1 are analyzed. Data associated with **(A-G)** are in S3 Data.
(EPS)

S17 Fig Comparing Dg RNAi and Scgδ RNAi. **(A-C)** Correlation circle plots for wild type, *Dg RNAi*, and *Scgδ RNAi* retinas for both secondary and tertiary pigment cells. **(D)** PCA of secondary and **(E)** tertiary pigment cells comparing *Dg RNAi* and *Scgδ RNAi* retinas. Correlation circle plots to analyze the correlation between each variable and the principal components and how well the variables explain the variance of the data (bottom panels). **(F, G)** Parameter comparisons extracted from the original data. The 2 parameters that contribute most to principal component 1 are analyzed. Data associated with **(A-G)** are in S3 Data.
(EPS)

S18 Fig Local Laminin accumulation is reduced upon expression of Sarcoglycanδ RNAi. **(A)** Basal surface of a 20h APF wild type LanA::GFP, and of a *Scgδ* RNAi LanA::GFP retina. Scale bar: 10 μm **(B)** Quantification of the LanA::GFP signal for these 2 genotypes. Data associated with panel **(B)** are in S3 Data.
(EPS)

S1 Data Excel file for graphs in Figs 3, S4 and S12. All measured shape parameters are given for each genotype, with each sheet labeled with the relevant genotype. These data were used to perform the PCA presented in Figs 3A–3C, S4D, S4E, S12A and S12D. These data were also used to generate the circle plots in S4A–S4E Figs and the box plots in S4F and S4G Fig.
(XLSX)

S2 Data Excel file for graph in Fig 4. Double normalized data for FRAP plotted in graph (4F). Normalized mean fluorescence intensity values for each indicated replicate (*y* axis) were calculated using the easyFRAP web application [91] averaged and then plotted against time (*x* axis). Raw fluorescence values for each replicate are included on a separate sheet.
(XLSX)

S3 Data Excel file for graphs in Figs 8, S13, S14, S15, S16 and S17. All measured shape parameters are given for each genotype, with each sheet labeled with the relevant genotype. These data were used to perform the PCA presented in Figs 8A–8C, 9A–9C, S13A, S13B, S14D, S14E, S15D, S15E, S16D, S16E, S17D and S17E. These data were used to generate the circle plots in S13A, S13B, S14A–S14E, S15A–S15E, S16A–S16E and S17A–S17E Figs, and also the box plots in S13C, S13D, S14F, S14G, S15F, S15G, S16F, S16G, S17F and S17G Figs.
(XLSX)

S4 Data Excel file for graph in Fig 8. Data for graph **(D)** is given in a sheet containing an image identifier, area and fluorescence intensity measurements, and the calculations used to determine the values for integrin enrichment (*y* axis) at the grommet in each genotype (*x* axis).
(XLSX)

S5 Data Excel file for graph in Fig 9. Data for graph **(E)** is given in a sheet containing an image identifier and fluorescence intensity measurements of Laminin antibody staining (*y* axis) in each genotype (*x* axis).
(XLSX)

S6 Data Excel file for graph in S18 Fig. Data for graph **(B)** is given in a sheet containing an image identifier and fluorescence intensity measurements of LanA::GFP levels (*y* axis) in each genotype (*x* axis).
(XLSX)

## Abbreviations

APF: after puparium formation
Dg: Dystroglycan
DGC: Dystroglycan receptor complex
ECM: extracellular matrix
Mew: Multiple edematous wing
Mys: Myospheroid
PCA: principal component analysis
RT: room temperature
